# Determination of Vancomycin Resistant *Enterococcus faecium *Diversity in Tehran Sewage Using Plasmid Profile, Biochemical Fingerprinting and Antibiotic Resistance

**DOI:** 10.5812/jjm.8951

**Published:** 2014-02-01

**Authors:** Katayoun Borhani, Ali Ahmadi, Fateh Rahimi, Mohammad Reza Pourshafie, Malihe Talebi

**Affiliations:** 1 Department of Microbiology, Pasteur Institute of Iran, Tehran, IR Iran; 2 Department of Microbiology, School of Medicine, Tehran University of Medical Sciences, Tehran, IR Iran; 3 Department of Biology, Faculty of Science, University of Isfahan, Isfahan, IR Iran

**Keywords:** *Enterococcus faecium*, Vancomycin Resistance, sewage

## Abstract

**Background:**

Sewage treatment plants are considered to be the hotspots for antibiotic resistance transfer among bacterial species. Many fecal bacteria including Enterococci circulate and are exposed to antibiotic residues in this environment. Being as one of the most common cause of nosocomial infections, special concerns have risen worldwide about the rate and characteristics of Enterococci (especially, isolates with high resistance against glycopeptides) which are available in raw sewages.

**Objectives:**

Study on the vancomycin Resistant *E. faecium *diversity in Tehran sewage by plasmid profile, biochemical fingerprinting and antibiotic resistance

**Materials and Methods:**

Forty isolates recovered from an urban sewage treatment plant were studied during 2009- 2010. The antibiotic resistance of isolates against 7 antibiotics was examined by disk diffusion method. Extraction of plasmid DNA was performed and identification of van genotype (*vanA *and *vanB*) was done by PCR. Biochemical fingerprinting was done by the use of Phene-Plate system (PhP).

**Results:**

All isolates were found to be resistant to erythromycin, ampicillin and ciprofloxacin. The PCR analyses showed that all *E. faecium* isolates harbored *vanA* gene and 5 (13%) isolates harbored *vanA* and *vanB* concomitantly. By plasmid profiling the VRE isolates differentiated into 11 types. PhP showed that VRE isolates were grouped into 23 biochemical types.

**Conclusions:**

The combination of plasmid profiling and PhP techniques revealed the presence of diverse population of VRE in sewage treatment plant in Tehran. Furthermore, the results showed that the PhP technique is a reliable method in determining the VRE clonal diversity.

## 1. Background 

Raw sewage contains many fecal bacteria which are naturally exposed to antibiotic residues and eventually become resistant to several antibiotics (1). Sewage treatment plants (STP) are considered to be the hotspots for antibiotic resistance transfer among bacterial species in the collected water from different sources (2). Enterococci are common microorganisms in environment where animal and human fecal materials are present (3). Over the past two decades, *Enterococcus *has been identified with an increasing frequency as the agent of nosocomial infections (4). At the same time, there has been a corresponding increase of antimicrobial resistance to most currently approved antibiotics among *Enterococcus* (2). 

In recent years there has been a special concern about the Enterococci with high resistance to the glycopeptide antibiotics such as vancomycin. Until now, this group of drugs has been the last option against multiresistant Enterococci infections (3). *VanA* phenotype, which is responsible for high resistance to both vancomycin and teicoplanin, is the most important phenotype among different types of vancomycin resistance (5). In contrast to *vanA* positive Enterococci, strains carrying *vanB* gene are not being associated to the disease outbreaks (5). 

As a result of a highly acquired antibiotic resistance, Enterococci are recognized as the main spreading agent of the vancomycin resistance in intra- and inter- enterococcal species (6). Since the antibiotic resistance genes involved in spreading of resistance, in part, are transferable via plasmids, Enterococci can contribute to the spread of antibiotic resistances in the environment (7). Although, plasmid profiling is very simple and rapidly performed, it is considered to have a low discriminatory power (8). Combination of plasmid profile, antibiotic resistance and biochemical fingerprinting could increase the ability to analyze the diversity of the isolated species. 

## 2. Objectives

Study on the diversity of vancomycin resistant *Enterococcus faecium* in Tehran sewage by plasmid profile, biochemical fingerprinting and antibiotic resistance.

## 3. Materials and Methods 

### 3.1. Sample Collection

Samples were collected three times from December 2009 to February 2010. The sampling was carried out in Ekbatan central STP located in west of Tehran. Samples were collected from urban raw sewages kept in sterile 250 mL bottles and transported to the laboratory on ice and analyzed within 3 hours.

### 3.2. Isolation of Enterococci

The samples were diluted 10-fold with phosphate-buffered saline and 200mL of diluted samples were filtered with 0.45μm filter membrane (Millipore Corporation, Bedford, MA, USA). The filters were put on brain heart infusion (Merck, KGaA, Darmstadt, Germany) agar and incubated at 37°C for 2 hours. Then the filters were transferred on Enterococcus agar (Becton Dickinson and Co., Sparks, MD, USA) containing 8 μg/mL vancomycin and incubated at 45°C for 48 hours (5). The filters were transferred to bile Esculin agar and incubated at 45°C for 2 hours. Black colonies were selected and biochemical tests including catalase, pyrrolidonylarylamidase (PYR) and growth on 6.5% NaCl condition (Merck, KGaA, Darmstadt, Germany) were performed. The isolates that grew under 6.5% NaCl and 45°C conditions, catalase negative and Esculin and PYR positive were defined as Enterococci (5).

These isolates were identified to the species level using the following biochemical tests; acid production of L-arabinose, lactose, D-sorbitol, D-mannitol, L-sorbose, methyl-α-D-glucopyranoside, arginine dihydrolase, motility, hippurate hydrolysis, haemolysis, pigmentation, and reduction of 0.04% tellurite (Merck, KGaA, Darmstadt, Germany) (9). 

### 3.3. Antimicrobial Susceptibility Test

Disk diffusion was done for seven common used antibiotics according to the CLSI guidelines (10). The following antibiotics were tested: ampicillin (10 µg), gentamicin (10 µg), vancomycin (30 µg), ciprofloxacin (5 µg), chloramphenicol (30 µg), erythromycin (15 µg), tetracycline (30 µg) (BBL, Sensi Disk, USA). The minimum inhibitory concentration (MIC) of the vancomycin and teicoplanin was evaluated by using E test (AB Biodisk, Sweden) (10). *E. faecalis *(ATCC 29212) and *E. faecalis *(ATCC 51299) were used as control strains.

### 3.4. Plasmid Extraction and PCR

Extraction of plasmid DNA was performed with a QIAprep Miniprep kit (Qiagen GmbH, Hilden, Germany) according to the manufacturer recommendations. Plasmid DNA separated by 0.8% agarose gel electrophoresis and stained with ethidium bromide. The patterns clustered by unweighted pair-group method with arithmetic averages (UPGMA) and Gelcompar II version 4.0 (Applied Maths, Sint-Matens-latem, Belgium). All VREF were tested for *van* genes using plasmid DNA. Identification of *van* genotype (*vanA* and *vanB*) for each isolate of VREF was performed by PCR with specific primers.

Primer sequences (*vanA*: 5'-CATGAATAGAATAAAAGTTGCAATA-3**', **5'-CCCCTTTAACGCTAATACGATCAA-3'*vanB*: 5'-GTGACAAACCGGAGGCGAGGA-3', 5'-CCGCCATCCTCCTGCAAAAAA-3') were derived from the published sequences of the genes (11). PCR assay was performed in a total volume of 25 µL containing 40 pM of each primer, 1.5 mM MgCl_2_, 0.2 mM of each dNTP,10 mM Tris-HCl (pH 8.3), 0.5 U of *Taq* DNA polymerase (HT Biotechnology, Cambridge, United Kingdom). The PCR was done using the following cycling condition: initial denaturation at 94° C for 3 minutes, 30 cycles of denaturation at 94° C for 1 minute, annealing at 54° C for 1 minute and extension at 72° C for 1 minute and a final extension at 72°C for 10 minute. The amplified PCR mixtures were identified by electrophoresis using 1% agarose gel and 90 V. The gels were then stained with ethidium bromide (11).

### 3.5. Biochemical Fingerprinting

For biochemical typing, the PhP-RF plate method (PhPlate AB, Stockholm, Sweden) was used. Then, eleven quantitative fingerprints were analyzed by PhPWin software version 4221 (PhPlate Microplates Techniques AB, Sweden) to assess the level of similarity (12). Preparation and inoculation of the plates was done according to the manufacturer instruction. The absorbance value (*A*_620_) of each well was measured at the 16, 40, and 64hours of incubation. The mean value of all three readings was calculated and the similarity value was measured as the correlation coefficient after pair wise comparison of the strains. After that, the similarity matrix was clustered according to the UPGMA to depict a dendrogram in which each isolate represents a horizontal line connected to each other at the similarity level. An identity level (ID level = 0.965) was set up by testing five isolates in duplicate. Isolates showing similarity level more than the identity levels were considered as identical (Common Biochemical Phenotypes: C-BPT). The diversity was defined as Simpson’s index of diversity (*D*_i_). The optical readings, calculation of correlation coefficients, diversity indexes and clustering all were done by the PhPWin software (12).

## 4. Results 

In all, 40 colonies were randomly selected from the colonies grown on *Enterococcus* agar and bile Esculin agar (BEA). PCR amplification was performed separately for each *vanA *and *vanB *genes. All of 40 VRE isolates carried *vanA* gene (100%) and 5 (13%) isolates harbored *vanA* and *vanB* genes, concomitantly.

Antibiotic susceptibility test showed that all VRE isolates (100%) were resistant to 5 antibiotics including ampicillin, erythromycin and ciprofloxacin, vancomycin and teicoplanin. Out of all isolates, 98% were resistant to gentamicin. Among the tested antibiotics, the lowest rate of resistance was found in chloramphenicol (20%) and tetracycline (15%). Overall, 4 different antibiotic resistance patterns were observed for all examined isolates (Table 1). 

**Table 1.** Antibiotic Resistance Pattern of 40 Vancomycin Resistant *E. faecium *Isolates

Pattern of Antibiotic Resistance	Isolates, No. (%)	Antibiotic Resistance Group
**Gm /Am/C/E/Cip^a^**	8 (20%)	1
**Gm /Am/E/TE/Cip**	6 (15%)	2
**Gm /Am/E/ Cip**	24 (60%)	3
**Am/E/Cip**	2 (5%)	4

^a^ Abbreviations: Am, ampicillin; C, chloramphenicol; Cip, ciprofloxacin; Gm, gentamicin; E, erythromycin; TE, tetracycline.

**Figure 1.**
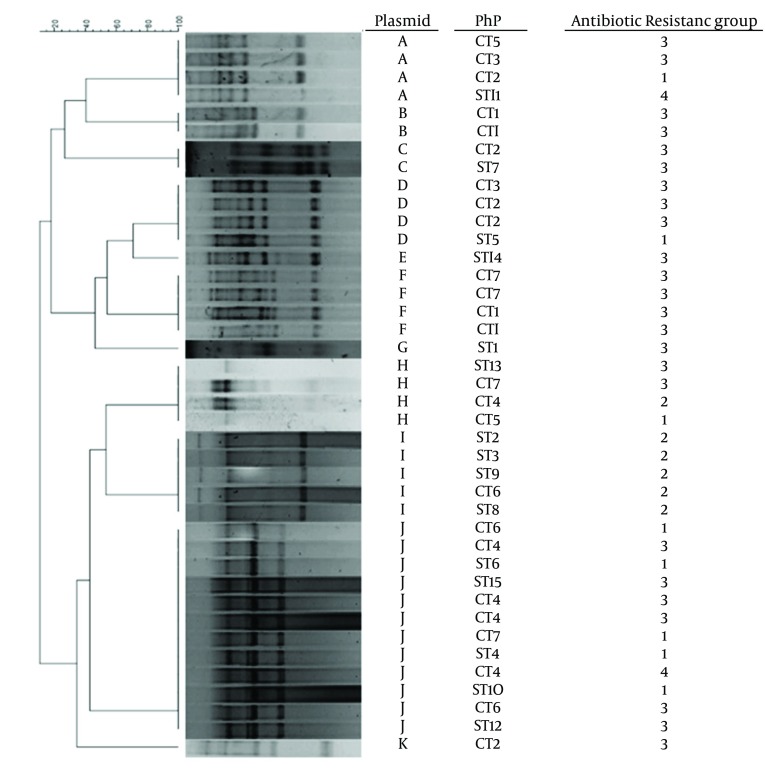
A UPGMA Dendrogram of 40 vancomycin Resistant *E. faecium *Strains Plasmid Profile UPGMA: Unweighted Pair Group Method with Arithmetic Mean

MIC of vancomycin showed that all of the isolates were highly resistant to vancomycin (MIC ≥128–256 µg/mL). The range of MIC for teicoplanin was 24-256µg/mL. The PhP typing of isolates revealed that all of our VRE isolates were from diverse populations (*D*_i_ = 0.96). Twenty-four isolates belonged to 7 common types and 16 isolates belonged to 16 single types. The common types were common in 2 to 5 isolates. According to plasmid profile, among 40 purified plasmids from VRE, 11 different plasmid profiles (A- k) were detected (Figure 1).

All of the isolates harbored plasmids. The plasmid profiles showed that the isolates differed from 2 to 9 bands. Three isolates (7.5%) showed distinct plasmid profiles (E, G, K) and the remaining 37 (92.5%) isolates showed identical plasmid patterns in 6 types comprising 2-12 isolates. The major cluster, J, contained 12 isolates with the same plasmid pattern. 

## 5. Discussion

Wastewater contains pathogens causing risks to human health unless measures are taken to control the hazard. The development of treatment process for sewage was driven by the need to reduce the environmental contamination because of uncontrolled discharge of human wastes to surface water (1). It is generally assumed that Enterococci are fecal bacteria with an excellent ability for adoption to adverse environmental conditions (13). In this study VRE were isolated from sewage at different occasions in Tehran. Recovery of sewages VRE and treated sewages has been shown by other studies (3). Other studies from European countries have reported a high prevalence of VRE in STPs (14-16). These studies reported that *E. faecium* was the most prevalent isolate followed by *E. hirae* and *E. faecalis *(17). In the contrary of other reports, *E. faecium *was the only VRE species isolated in present study. 

The results of antibiotic susceptibility tests revealed that all or most of our isolates were resistant to 5 or 6 antibiotics which are commonly used in the clinical wards. Our results confirmed the reported data that previously published by other authors, indicating that *E. faecium *are frequently and increasingly becoming resistance to multiple antibiotics (18). On the basis of biochemical fingerprinting and plasmid profiles, 23 PhP types with discriminatory power of (Di = 0.96) were determined. By using plasmid profiling alone, 11 patterns out of 40 isolates of VRE were identified. The presence of diverse plasmid and biochemical fingerprinting patterns amongst the VRE isolates suggest the dissemination of the *vanA* gene cluster among different strains of *E. faecium.*

Although plasmid analysis is not the method of choice for fingerprinting, it could provide useful epidemiology information. To some extent, it is useful for determining the bacterial potential for transmission of the antibiotic resistance (19). Our results showed that some isolates such as plasmid types B strain had an identical biochemical characteristics and antibiotic resistance patterns. On the other hand, the isolates with identical plasmid profile (i.e. A, H or J) had different PhP and antibiotic resistance pattern. There could be several reasons for this: i) the antibiotic resistance was developed by other mode besides conjugation, ii) following conjugation and plasmid transfer certain mutation have occurred within the isolated species that resulted in antibiotic resistance or iii) the isolates were originally resistant to the selected antibiotics.

The result of this study revealed that *vanA* positive *E. faecium* is ubiquitous in STPs of Tehran. The presence of different VRE clones in the sewage suggests the ability of these bacteria in disseminating the resistance genes. Consequently, reducing the release of bacteria or genetic elements to the community is becoming a critical issue to avoid the increase of environmental reservoirs of antibiotic resistance. Furthermore, these results indicated that plasmid typing in combination with PhP could be suitable techniques for studying on VRE clone types. 
